# A practical approach to communicating benefit-risk decisions of medicines to stakeholders

**DOI:** 10.3389/fphar.2015.00099

**Published:** 2015-06-11

**Authors:** James Leong, Stuart Walker, Sam Salek

**Affiliations:** ^1^Center of Regulatory Excellence, Duke-NUS Graduate Medical SchoolSingapore, Singapore; ^2^Centre for Innovation in Regulatory ScienceLondon, UK; ^3^Department of Pharmacy, School of Life and Medical Sciences, University of HertfordshireHatfield, UK; ^4^Institute for Medicines DevelopmentCardiff, UK

**Keywords:** benefit–risk assessment, benefit–risk methodologies, pharmaceutical industry, regulatory agency, framework, documentation

## Abstract

**Purpose:** The importance of a framework for a systematic structured assessment of the benefits and risks has been established, but in addition, it is necessary that the benefit-risk decisions and the processes to derive those decisions are documented and communicated to various stakeholders for accountability. Hence there is now a need to find appropriate tools to enhance communication between regulators and other stakeholders, in a manner that would uphold transparency, consistency and standards.

**Methods:** A retrospective, non-comparative study was conducted to determine the applicability and practicality of a summary template in documenting benefit-risk assessment and communicating benefit-risk balance and conclusions for reviewers to other stakeholders. The benefit-risk (BR) Summary Template and its User Manual was evaluated by 12 reviewers within a regulatory agency in Singapore, the Health Sciences Authority (HSA).

**Results:** The BR Summary Template was found to be adequate in documenting benefits, risks, relevant summaries and conclusions, while the User Manual was useful in guiding the reviewer in completing the template. The BR Summary Template was also considered a useful tool for communicating benefit-risk decisions to a variety of stakeholders.

**Conclusions:** The use of a template may be of value for the communicating benefit-risk assessment of medicines to stakeholders.

## Background

Regulators are challenged to review the overall balance between the benefits and the associated risks of new drugs rather than the impact of individual components (Breckenridge, 2010). In this context, the key components for effective regulation are transparency and accountability underpinned by the use of a structured framework that aids in the communication of the differences in opinion between regulators and the drug developers (World Health Organization, 2003). Commenting on innovations in regulatory science, Leufkens and Eichler suggested that there are three dimensions in this area. First, regulators should keep current their understanding of the science and technologies that help in drug development and the advancement in innovation. Second, new standards and tools should be developed to evaluate and assess the benefit-risk balance of medicines to facilitate a sound and transparent decision-making process. Last, the entire system should be monitored for its impact on patient safety, public health and meeting medical needs (Leufkens and Eichler, 2011). Therefore, it is likely that a new overarching framework would be required to encompass these new initiatives.

In the European Medicines Agency's (EMA) *Roadmap to 2015*, one of the strategic areas identified was facilitating the access of medicines through reinforcing the benefit-risk balance assessment model, to be achieved through a set of priority activities. These included looking at appropriate quantitative tools, improving the quality and consistency of the outcomes, reviewing the European Public Assessment Reports (EPARs) to improve communication of benefit-risk decisions to stakeholders and increasing the involvement of patients, academia and healthcare professionals in the assessment of medicines to ensure their views are taken into consideration (European Medicines Agency, 2011).

EMA proposed PROACT-URL (Problem formulation, Objectives, Alternatives Consequences, Tradeoffs, Uncertainties, Risk tolerance, Linked decisions), a benefit-risk framework that consists of eight steps (European Medicines Agency, 2010), and beginning in 2009, the US Food and Drug Administration (FDA) have taken initiatives to explore systematic approaches to assess and communicate benefits and risks (Centre for Innovation in Regulatory Science, 2011; US Food and Drug Administration, PDUFA, 2012; US Food and Drug Administration, 2013). In addition, over the next few years, the Therapeutic Goods Administration (TGA) of Australia will be focusing on increasing transparency and engaging stakeholders with a new framework for communication that includes the benefits vs. risks approach in their regulation of medicine (Therapeutic Goods Administration, 2013).

At the same time, the pharmaceutical industry has been taking the initiative to address the need for an improved benefit-risk assessment of medicines by developing a structured, systematic, and transparent framework. The Pharmaceutical Research and Manufacturers of America (PhRMA), the Benefit-Risk Action Team (BRAT) Framework sought to incorporate all the relevant aspects of benefits and risks in drug development in both qualitative and quantitative analyses (Coplan et al., 2011; Levitan, 2012).

**Figure 1:**
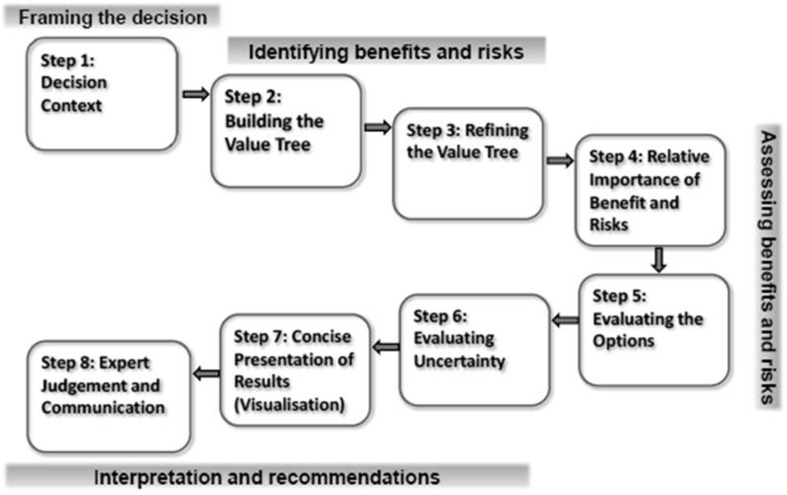
**The UMBRA framework**. Reprinted with permission, Centre for Innovation in Regulatory Science.

Leong and colleagues explored need for a universal benefit-risk framework for medicines within regulatory agencies and pharmaceutical companies and the status of its development (Leong et al., 2013). The advantages of a universal framework reported by both these groups were similar to those mentioned earlier, while the main hurdle to its establishment was the lack of an accepted, validated, and international model. Although stakeholders are looking forward to a change, the benefit-risk system that is likely to be adopted is an overarching, semi-quantitative framework that incorporates a toolbox of various benefit-risk methodologies. As a response to this ongoing need, a workshop was organized that brought together regulators and the pharmaceutical industry, where the attendees agreed the common elements of an overarching, internationally acceptable, standardized benefit-risk framework (Figure 1), known as the Universal Methodologies for Benefit-Risk Assessment (UMBRA) (Centre for Innovation in Regulatory Science, 2012).

In collaboration with the Centre for Innovation in Regulatory Science (CIRS), TGA, the Singapore's Health Sciences Authority (HSA), Health Canada and Swissmedic, a Consortium on Benefit-Risk Assessment (COBRA), was formed that aimed to develop a systematic qualitative approach for the benefit-risk assessment of medicines in order to facilitate joint or shared reviews by these four agencies. A benefit-risk documentation template (BR Template), was developed based on the EMA reflection paper on benefit-risk assessment (European Medicines Agency, 2008) and reviewed by the Consortium through both retrospective and prospective studies employing its use, with plans for making the template more reflective of actual practice (Centre for Innovation in Regulatory Science, 2012).

Notwithstanding recent initiatives by both EMA (European Medicines Agency, 2008, 2010) and US FDA, there is currently no standard template for the documentation and communication of the evaluation outcomes of benefit-risk decisions. Hence there is a need to find appropriate tools to enhance communication in a manner that would uphold transparency, consistency and appropriate standards. It was proposed therefore, that a summary version of the full BR Template that had been developed and evaluated by COBRA, which could collate the relevant conclusions leading to the final benefit-risk decision, might also be useful in documenting and communicating benefit-risk decisions for the emerging markets. Hence this study aimed to determine the value of the use of a summary template in documenting the benefit-risk assessment of medicines during the review and communicating benefit-risk balance and conclusions, among regulators in the emerging markets and also to other stakeholders including the industry and health technology assessment agencies. In addition, the effectiveness of a user manual in guiding a reviewer to complete the summary template was also evaluated.

## Materials and methods

This research was conducted as a retrospective, non-comparative study at the HSA Singapore, an established regulatory agency from the emerging markets. The BR Template used by the Consortium was reviewed and its *Benefit-Risk Summary* section was extracted to produce the BR Summary Template (Supplementary Material). A User Manual was also created as was the study evaluation tool to review the uses of both BR Summary Template and User Manual. The overall study package including the study protocol, BR Summary Template, User Manual (Supplementary Material) and the study evaluation tool (Supplementary Material) was sent to 16 clinical reviewers in HSA involved in assessing the benefit-risk balance and registration of medicines. The reviewers were asked to identify an appropriate product application based on the following criteria:

- A new drug application that requires a benefit-risk evaluation;
- An abridged review, applicable to products having obtained a marketing approval in at least one country;
- A regulatory decision (having received marketing approval or a confirmed benefit-risk decision) obtained within the last 3 months.

The reviewers transferred the relevant information required for the BR Summary Template from their respective completed clinical assessment reports (as per current processes in HSA) with the support of the User Manual. Following this transfer, the reviewers completed the study evaluation tool. The evaluation was to assess the entire experience of using the BR Summary Template. All responses were collated into a single group and the outcomes are presented according to their respective sections in the study evaluation tool. All data were expressed as percentage over number of respondents for that item. Free-text comments were collated and presented in appropriate categories where necessary. This was designed as an exploratory and descriptive study and the outcomes were interpreted to provide qualitative inferences relating to the aims. This was an exploratory qualitative study and therefore no statistical analyses were planned or conducted. The results were displayed using descriptive statistics.

## Results

A total of 12 responses were received by August 2013. Of the four who did not respond, one was transferred to another unit, two did not have applications that met the criteria and the remaining one did not respond. Nine of the respondents had between 1 and 5 years of working experience in the agency, with one having less than 1 year and two having more than 5 years. As the reports were written independently, the responses actually represented the evaluation of 10 different products reviewed via the abridged route.

### Part I—user-friendliness of the BR summary template and the user manual

The template has two functions to help users navigate the document, namely the “Go to page” button and page thumbnails to locate a specific page. These are aimed at reducing the effort required to move between different sections. The “Go to page” button appeared to be the more useful, as 10 reviewers rated it either good or excellent. Seven respondents indicated that the page thumbnails were fair or were not used and it was commented that the thumbnail icons were too small to decipher the contents and bookmarks might have been more effective, although none rated the BR Summary Template as not user-friendly. There was a suggestion to include a “Back” button to the content page or another primary page.

The User Manual was provided to guide the reviewer on the steps to complete the template, as well as to clarify the common terms used in the template. Between 9 and 12 respondents rated the clarity, comprehensiveness and applicability of the User Manual as “good.”

None rated the manual as poor for any of the three parameters. Comments received included the suggestion to provide examples or a case study in the manual to better illustrate the use of the template. It was also concluded that an inexperienced reviewer might find the manual insufficiently comprehensive. Even though the User Manual provided instructions with regard to assigning relative importance to benefit and risk parameters, the lack of experience by the reviewers may have prevented them from effectively completing the BR Summary Template in this aspect.

### Part II—appropriateness (fit for purpose) of the documentation of the BR summary template

**Figure 2:**
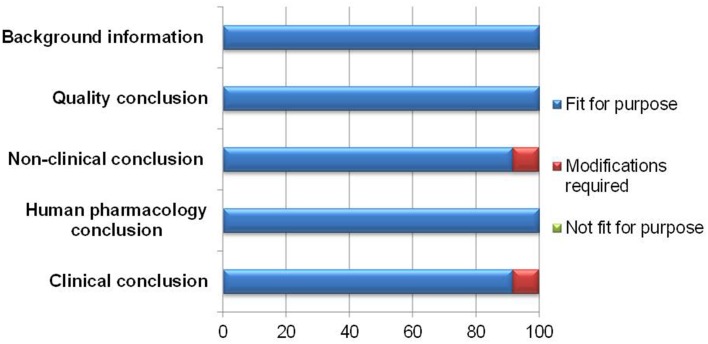
**Documentation of relevant information supporting the benefit-risk decision**.

The appropriateness of a template is dependent on its capability to present the processes leading to the final benefit-risk conclusion in a structured and systematic manner. In documenting the various conclusions, the BR Summary Template was largely thought to be fit for purpose by 11–12 respondents (Figure 2).

One modification suggested was to clarify the difference between the clinical conclusion section and the overall conclusion for benefit-risk balance, as it might appear redundant if misunderstood. The other modification recommended was to make more guidance on writing the non-clinical conclusion available, as some reviewers were not familiar with providing details for this section.

**Figure 3:**
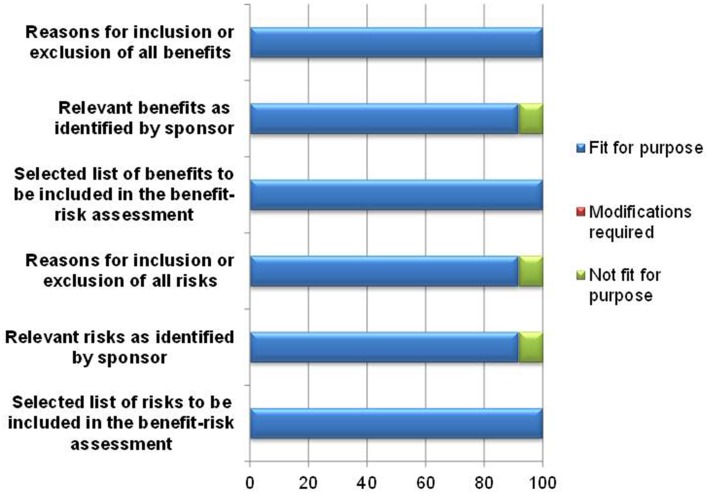
**Documentation of benefits and risks**.

The template's ability to document the benefits and risks of the product being evaluated was affirmed by 11–12 of the respondents (Figure 3). One respondent was unsure of the usefulness of the template in documenting relevant benefits and risks as identified by the sponsor and rated these parameters as not fit for purpose, as the reviewer would eventually indicate the benefits and risks that are to be included for assessment. However, the purpose of listing benefits identified by the sponsor and the reasons for inclusion or exclusion by the reviewer in the template is the transparent and full provision of the rationale for the benefit-risk decision. Another respondent felt that there must be greater clarity in the template in defining risks considered critical to the benefit-risk assessment, and as a result this reviewer rated the documentation of inclusion or exclusion of all risks as being not fit for purpose.

**Figure 4:**
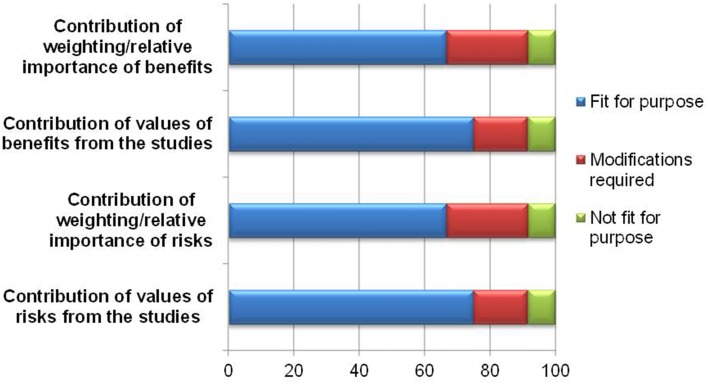
**Documentation of weights and values**.

The exercise of indicating relative importance and numerical values in the identified benefits and risks is aimed at improving the articulation of the basis of the benefit-risk decision. While the majority of the respondents believed it was fit for purpose, 3–4 felt that the template required modifications or was not fit for purpose (Figure 4). The reasons and comments are listed in Table 1 and can be seen as proposed amendments to the template to improve documentation of weights and values. It can be concluded that the lack of understanding of weighting and valuing in general is the root cause of the above observation.

**Table 1:** **Amendments required to improve the documentation of weights and values**.

**Modifications required**
Clarification on how to assign weights
Provide more instructions on how to complete these sections on weighting and valuing
Recommend a consistent approach for weighting through a drop-down list of either numerical ranking or qualitative descriptors
Recommend a free text box for cases whereby the weightings are not clear-cut
Clarify if the weightings are to add up to 100% for both the benefits and risks, or are they to be considered separately for each component
Provide some examples to illustrate the intention of the sections
**Reasons not being fit for purpose**
Not sure how to complete these sections

**Figure 5:**
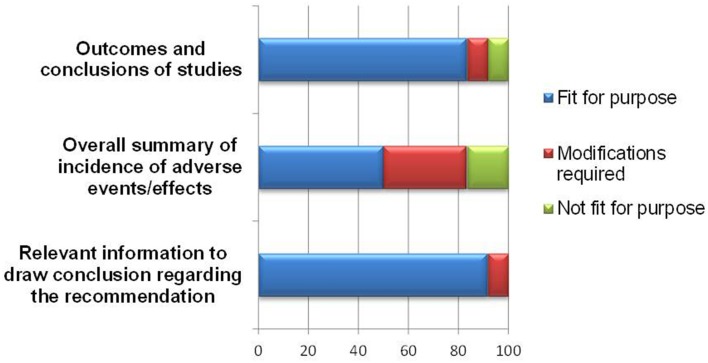
**Documentation of study outcomes, safety information and overall conclusion**.

Overall, the BR Summary Template appeared to be able to document study outcomes and relevant benefit-risk information leading to a regulatory recommendation, with 10–11 of respondents agreeing on this (Figure 5).

With regards to documenting study outcomes, one respondent recommended modification to allow for applications based on bibliographic submission or published literature. Another respondent who rated the template “not fit” for presenting study outcomes commented that this section did not contribute to the overall benefit-risk assessment. As for the template being useful in presenting information leading to a regulatory recommendation, one respondent indicated that more clarification on weighting should be provided in order to achieve this purpose.

As for presenting an overall summary of the adverse events or effects, half of the respondents felt that either a modification was required, or the template was “not fit” for this purpose. The amendments required are listed in Table 2 and are largely technical in nature to accommodate other formats for uploading safety information.

**Table 2:** **Amendments required to improve the documentation of overall summary of adverse events and effects**.

**Modifications required**	**Reasons being not fit for purpose**
Allow text format, PDF snapshots or other common formats besides the picture formats	This section does not serve the overall benefit-risk assessment
Further categorization to listing of common treatment-emergent AEs, serious AEs, death, discontinuations, etc.	Difficulties in attaching the PDF file
	As the studies had different safety endpoints, there was no pooled summary

### Part III—applicability of the BR summary template

**Figure 6:**
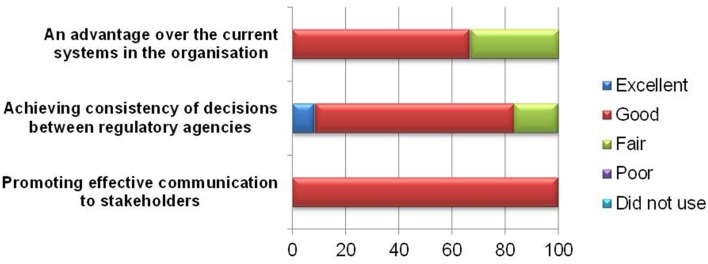
**Applicability of BR Summary Template**.

The primary goal of the BR Summary Template is to communicate regulatory decision making to internal or external stakeholders. All the respondents found the template effective in promoting communication to stakeholders (Figure 6), and 10 of respondents believed it could help achieve consistency of decisions between regulatory agencies. However, one respondent commented that with the different weightings applied, consistency in regulatory decisions across agencies cannot be achieved.

Four respondents felt that the template did not confer any additional advantage over the current processes in the organization. For new users, this approach generally appears more difficult to use than the current HSA report template as the system in use is more efficient and reaches the same conclusion. Incidentally, the BR Summary Template is a repeat of a section of the existing HSA current report template. Moreover, the BR Summary Template was formatted as a PDF, which makes the use and uploading of information more tedious compared with the existing Word document.

**Figure 7:**
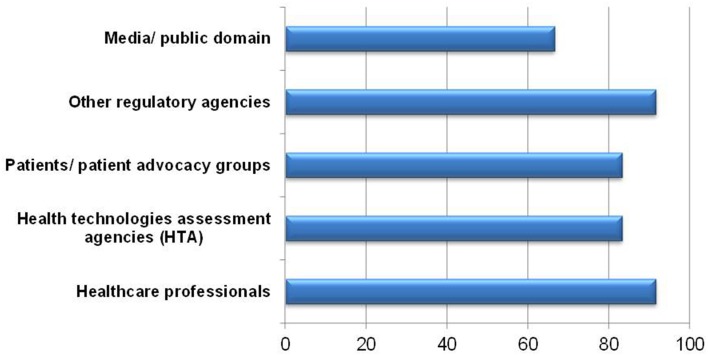
**Willingness to share the BR Summary Template with various stakeholders**.

When asked, 11 of respondents were willing to share the completed BR Summary Template with healthcare professionals and other regulatory agencies (Figure 7). One respondent indicated sharing with health technology assessment (HTA) agencies was not applicable since HTA agencies are not currently in use in Singapore. One respondent commented that the template could not adequately describe the benefit-risk findings. Reservations in sharing with patients, patient advocacy groups, media and in public domains included the use of technical terms and medical jargon being unsuitable for lay persons, which may lead to confusion and misinterpretation. This challenge in understanding could invite unnecessary criticism and one respondent suggested that only selected sections be made available to such stakeholders.

### Part IV—general amendments to the BR summary template

One reviewer suggested combining the identification of benefits and risks with the exercise of assigning weights and values to avoid repetition. However, this suggestion could be accommodated by auto-populating the benefits and risks in Section Results of the template into Section 6 (Supplementary Material). It was commented that more guidance could be given on listing the reasons for inclusion or exclusion of benefits, such as local disease burden, available alternatives, strength of evidence, clinical relevance and convenience to patients. For completeness, one reviewer recommended adding another section to indicate if the benefit-risk balance is positive or negative, before being asked to provide reasons for a negative benefit-risk balance. While this study was conducted for new active substances, one reviewer recommended that the template could be amended to accommodate clinical variations.

## Discussion

The findings of this study showed that the successful implementation of a new process or tool in an established regulatory agency is dependent on the fundamental understanding of the principles behind their use. Weighting and valuing is seen as an explicit presentation of the subjective interpretation of clinical information. This exercise aimed to enhance the transparency of decision making by making it clear that the priorities placed on a set of benefits and risks ultimately affect the resulting benefit-risk balance. However, the concept of weighting or assigning relative importance and valuing is a technique that is relatively new to both HSA and possibly other regulatory agencies (Leong et al., 2013). Without an understanding of the rationale behind weighting and valuing, some reviewers could not appreciate its contribution to effective documentation and communication.

For improved use, the current BR Summary Template would require a revision to the technical capabilities and an improvement for the documentation of safety information and adverse events. In addition, the User Manual should be revised to include examples and case studies to better illustrate the use of the template. However, it appears that the capacity of the BR Summary Template to effectively communicate a benefit-risk decision has been clearly exhibited, as supported by the reviewers who were willing to share this template with stakeholders.

It is also appropriate to examine the utility of this template as a means of transferring knowledge and communicating the basis of a decision and the reviewers in this study indicated their willingness to share the completed BR Summary Template for a specific product with other regulatory agencies where there is a memorandum of understanding. For major regulatory agencies it may be a requirement to provide details of the evaluation to achieve a level of transparency stipulated by the jurisdiction. However, this study has demonstrated that the BR Summary Template, even in the absence of these details, is an effective tool to communicate benefit-risk decisions. Therefore, it may be considered as a basic report template for agencies that are in transition to build up their evaluation capabilities and would be an ideal tool for communicating benefit-risk decisions to regulatory agencies in jurisdictions with emerging pharmaceutical markets, since the components of the template address the basic needs of a sound and scientific discussion. Indeed, for regulatory agencies in emerging markets that are more resource constrained with respect to their scientific capabilities, the BR Summary Template may serve as a template for the assessment of medicines and as an internal standard in their pursuit to develop the capabilities of their agencies. There should be further studies to assess the use of the BR Summary Template in aiding emerging markets in their pursuit of improving their regulatory standards. This is in line with the earlier findings from a CIRS workshop to include the emerging markets earlier in the development of benefit-risk frameworks, so as to increase the worldwide acceptance of a universal framework (Centre for Innovation in Regulatory Science, 2008). It is only through a global understanding of the need for a common template that consistency in evaluating benefits and risks can be achieved. In addition, a fuller understanding of the utility of the tool could be obtained through further studies to examine the value of the tool in communicating benefit-risk decisions to healthcare professionals, patients and other stakeholders.

The limitations of this study were namely small sample size, single emerging market and the non-responders. However, the value of the study largely lies with the establishment of the applicability and practicality of such methodology which was introduced to the region for the first time. Nevertheless, the findings of the study and the use the BR Summary Template and the User Manual by the HSA reviewers proved to be useful, but would benefit from further validation studies.

The outcome of this study, involving reviewers within HSA Singapore as representative of the emerging markets in the region, has demonstrated that the principles of the BR Summary Template may be applicable to other jurisdictions or similar agencies, further evidence, however, from other countries in the region implementing the BR Summary Template should support this claim. This is indeed encouraging in the current climate, where the debate surrounding the benefit-risk assessment of medicines is on the top of many regulatory agencies' agenda. Thus, the promising features of the BR Summary Template will, no doubt, contribute to such on-going discussion.

## Author contributions

JL conceived the study, participated in its design and coordination and helped to draft the manuscript. SW conceived the study, participated in the study design and coordination and helped to draft the manuscript. SS conceived the study, participated in the study design and coordination and helped to draft the manuscript.

### Conflict of interest statement

All authors declare no payment or service from a third party for any aspect of the submitted work; no financial relationships with any entities that could be perceived to influence, or that give the appearance of potentially influencing what was written in the submitted work; no patents, copyrights or royalties relevant to the work; and no other relationships or activities that readers could perceive to have influenced or that give the appearance of potentially influencing what was written in the submitted work. The authors declare that the research was conducted in the absence of any commercial or financial relationships that could be construed as a potential conflict of interest.
