# Supplementary material for: A practical approach to communicating benefit-risk decisions of medicines to stakeholders
Source: Front Pharmacol. 2015 Jun 11;6:99. doi: 10.3389/fphar.2015.00099 (PMC4463867; doi:10.3389/fphar.2015.00099)
Supplement: Supplementary file 1 [file Presentation1.PDF]

Appendix A

Print Summary

Summary Template for the Benefit-Risk Assessment of Medicines

Participant(s):

....

Compound Identifier(s):	
Product name/ Brand name / Generic name:	
Active Ingredient(s)/ Strength(s)/ Dosage form:	
Proposed Indication:	

<u>Please complete a new summary form for each indication</u>

All data will be treated in strict confidence.
No data or information will be revealed to any third party

The UMBRA Eight Step Benefit Risk Framework

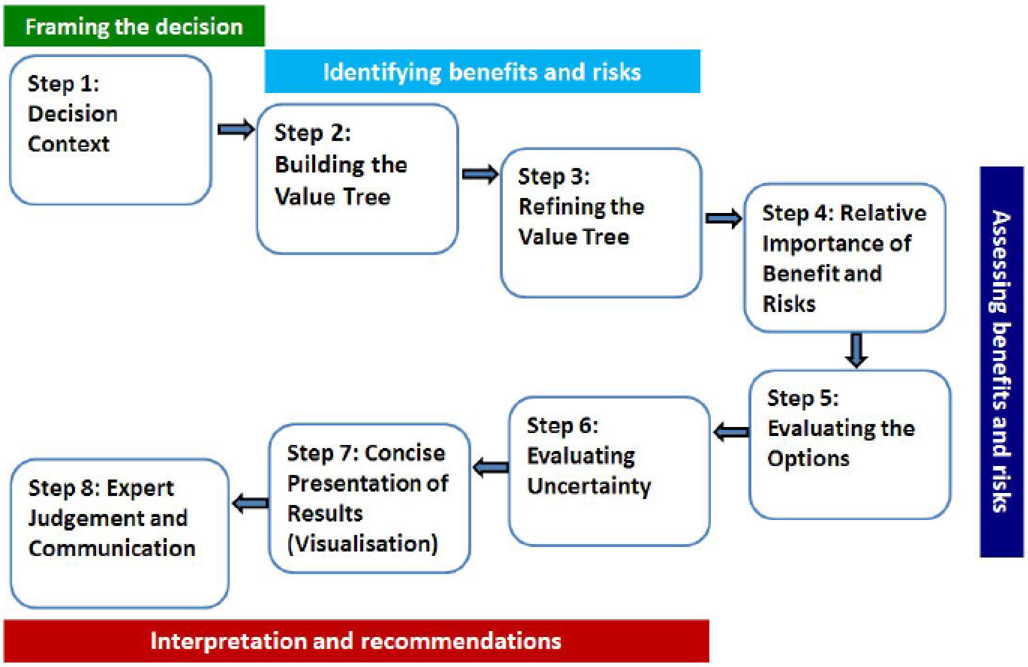

The diagram shows the common elements of the UMBRA eight step Benefit Risk Framework that make up a systematic approach to benefit-risk assessment for medicines

At the CIRS annual workshop, 2012 (20-21 June) there was a consensus from those who are developing Benefit Risk methodologies for assessing medicines that there are four key stages namely;

- Framing the decision;
- Identifying the benefits and risks;
- Assessing the benefits and risks;
- and Interpretation and recommendation.

Underpinning these was an overarching eight step framework;

1. Discision context;
2. Building the Value Tree;
3. Value Tree refinement;
4. Assessing relative importance;
5. Evaluating options;
6. Evaluating uncertainty;
7. Concise presentation of results – visualisation;
8. Final recommendation.

All the methodologies currently being developed by regulators and companies have these steps whether explicitly or implicitly undertaken.

The UMBRA overarching framework provides the basis for a common agreement on the principles for benefit risk assessment of medicines.

Table of Contents

Benefit-Risk Summary		
1.1	Background (Decision Context)	Go to Page
2.1	Overall Summaries	Go to Page
3.1	Identified Benefits and Risks	Go to Page
4.1	Clinical Study Summary	Go to Page
5.1	Table of Pooled overall Incidence of events	Go to Page
6.1	Relative Importance and Values	Go to Page
7.1	Conclusion	Go to Page

BENEFIT RISK SUMMARY:

This section provides a summary of the key outcomes of Benefit Risk analysis undertaken.

Summary 1.1 Background (Decision Context):

Summary 1.1.1 Specify the proposed therapeutic indication

Summary 1.1.2 Treatment modalities evaluated in this submission

Summary 1.1.3 Is this product for an unmet medical need? Please select

Reason:
Please provide justification for your decision on the product fulfilling or not fulfilling an unmet medical need

Summary 2.1 Overall Summaries:

Summary 2.1.1 Quality Conclusion:

If box ticked - No relevant findings for the clinical benefit-risk assessment ☐

If there are relevant findings please comment

Summary 2.1.2 Non-Clinical Conclusion:

If box ticked - No relevant findings for the clinical benefit-risk assessment ☐

If there are relevant findings please comment

Summary 2.1.3 Human Phamacology Conclusion:

Only the important results and issues that have an impact on the benefit-risk balance should be described. In addition, unresolved issues or uncertainties should be identified and their impact on the balance assessment should be clearly stated. This includes Bioequivalence, Pharmacokinetic and Dynamic profile, as well as PK, & PD interactions, special populations, dose findings etc.

Summary 2.1.4 Clinical Conclusion:

Only the important results and issues that have an impact on the benefit-risk balance should be described. In addition, unresolved issues or uncertainties should be identified and their impact on the balance assessment should be clearly stated. This includes study design, dosage, population and comparators.

BENEFIT RISK SUMMARY CONT:

Summary 3.1 Identified Benefits and Risks

Summary 3.1.1 Benefits documented

List all **benefits** of treatment for this indication as inferred in the submission	Please tick here if Benefit Identified by Reviewer but not by company	Please indicate which **benefits** you believe are justified to be included in the benefit risk assessment by ticking the box	Please explain your main reason **for inclusion or exclusion** of the benefit parameter
	☐	☐	
	☐	☐	
	☐	☐	
	☐	☐	
	☐	☐	
	☐	☐	
	☐	☐	
	☐	☐	
	☐	☐	
	☐	☐	
	☐	☐	
	☐	☐	
	☐	☐	
	☐	☐	
	☐	☐	

<u>BENEFIT RISK SUMMARY CONT:</u>

Summary 3.1.2 Risks documented

List all **risks** of treatment for this indication as inferred in the submission	Please tick here if Risk Identified by Reviewer but not by company	Please indicate which **risks** you believe are justified to be included in the benefit risk assessment by ticking the box	Please explain your main reason **for inclusion or exclusion** of the risk parameter
	☐	☐	
	☐	☐	
	☐	☐	
	☐	☐	
	☐	☐	
	☐	☐	
	☐	☐	
	☐	☐	
	☐	☐	
	☐	☐	
	☐	☐	
	☐	☐	
	☐	☐	
	☐	☐	
	☐	☐	

Summary 4.1 Clinical Study Summary

Study Ref. Type	Study Design (N)(duration) R, C, DB, OL (N=)(weeks/months) ·Non-inferiority/Superiority/ Observational study ·State primary objective ·State primary efficacy parameter	Treatment ·Treatment arm Active (name, dose, freq, duration) ·Comparator arm Placebo / Active (name, dose, freq, duration)	Conclusion ·Results of primary efficacy parameter ·Results of other relevant efficacy endpoints ·Conclusion of study (outcomes, strength of study, weight of evidence, and clinical significance)	
				-
				-
				-
				-
				-

Legend
R: Randomised **C:** Controlled **DB:** Double blinded **OL:** Open label **N:** Number of subjects

Click to add a study +

Summary 5.1 RISKS: Overall Summary

Table of pooled overall incidence of events can be added below

Adobe **Acrobat** users can click here to attach a file: Attach a file *(Note: this will not activate in Adobe **Reader**)*

Click in the space below to upload an image: (jpeg, gif, png): *(Available to both Adobe Reader and Acrobat users)*

Note: Click on an image to change it for another. To delete the image click 'Remove Table'.
You may need to add another table first as there must always be at least one table.

Remove table | Add another table

BENEFIT RISK SUMMARY CONT:

Summary 6.1 Weights and values

Benefits	Relative Importance (weighting)	Valuing the options			Comment on strength and uncertainty of benefit
		Investigated product	Comparator	Placebo	

Please describe methodology used for assessing relative importance: eg Ranking or point allocation and also what is has been used in relation to valuing the options e.g. % change, Number of patients, etc

BENEFIT RISK SUMMARY CONT:

Risks	Relative Importance (weighting)	Valuing the options			Comment on strength and uncertainty of each risk	Was the value or weight of this risk altered or mitigated by the ability to control the use of the medicine once on the market?
		Investigated product	Comparator	Placebo		

Please describe methodology used for assessing relative importance: eg Ranking or point allocation and also what is has been used in relation to valuing the options e.g. % change, Number of patients, etc

BENEFIT RISK SUMMARY CONT:

Summary 7.1 Conclusion

**Summary 7.1.1** If the benefit-risk balance is assessed to be negative, describe the harm (e.g. in terms of lack of efficacy, toxicity) that the drug may cause if used in the proposed indication
**Summary 7.1.2** Describe how the benefit-risk balance is expected to evolve over time (e.g. when late side effects emerge or long-term efficacy decreases)
**Summary 7.1.3** Describe outstanding issues, and other significant information eg, submission of additional reports by the company to address those issues, hearings and advisory group recommendations, information from other jurisdictions (eg advisory committees, scientific experts, patients, consumers, consumer advocates and other stakeholders)
**Summary 7.1.4** Make reference to the evaluation of the pharmacovigilance plan and risk minimization plan if any. Describe any communication or particularly significant information to the medical profession, patients or the public that is required. Describe restrictions to product availability or usage
**Summary 7.1.5** Describe the need for further studies (e.g. the need for studies to improve the benefit-risk balance with further optimization studies, the need for intensive additional follow up measures or specific obligations, and the need for further development including any paediatric development plans.

Summary 7.1.6 Please provide any other information considered by the agency relevant to the benefit risk decision that is not covered elsewhere in the proforma.

Summary 7.1.7 Please provide a clear conclusion on the benefit-risk being positive or not for the proposed indication.

Summary 7.1.8 Please provide the indication recommended following the outcome of the benefit-risk balance.

Reviewers Name:

Signature:

Date:

Manager sign-off or Peer review

Reviewers Name:

Signature:

Date:
