# Supplementary material for: A practical approach to communicating benefit-risk decisions of medicines to stakeholders
Source: Front Pharmacol. 2015 Jun 11;6:99. doi: 10.3389/fphar.2015.00099 (PMC4463867; doi:10.3389/fphar.2015.00099)
Supplement: Supplementary file 2 [file Presentation2.PDF]

Appendix B

Summary Template for the Benefit-Risk Assessment of Medicines

Professor Sam Salek,
Dr. James Leong and Professor Stuart Walker

User Manual

Table of Contents

Section	Page No.
Introduction	3
Glossary	3
Completing the Template – Cover page	6
Completing the Benefit Risk Summary Template	
• Summary 1.1 Background (Decision Context)	8
• Summary 2.1 Overall Summaries	9
• Summary 3.1 Identified Benefits and Risks	11
• Summary 4.1 Clinical Study Summary	12
• Summary 5.1 Risks: Overall Summary	13
• Summary 6.1 Weights and values	14
• Summary 7.1 Conclusion	15
Navigating through the template	17
Appendix A	18
Appendix B	19
Appendix C	20

Introduction

This manual has been developed as an aid for the user in completing the Summary template. It provides guidance to the user on how to complete the template, through understanding the terms used in the glossary and clarifications offered at various sections.

Throughout this manual, a red arrow “ 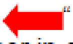 “ will be used to indicate sections where additional clarifications are provided to guide the user in completing the template.

Glossary

Term	Definition
Adverse event*	Also known as Adverse experience, it is any untoward medical occurrence in a patient or clinical investigation subject administered a pharmaceutical product and which does not necessarily have to have a causal relationship with this treatment.
Adverse reaction/effect*	In the pre-approval setting when the therapeutic dose(s) may not be established, it is all noxious and unintended responses to a medicinal product related to any dose should be considered adverse drug reactions.<br><br>For marketed medicinal products, it refers to a response to a drug which is noxious and unintended and which occurs at doses normally used in man for prophylaxis, diagnosis, or therapy of disease or for modification of physiological function.
Benefit	A potential favourable effect seen to be promoting or enhancing the current state of health, resulting from the treatment using the Product**
Benefit-risk assessment	Also referred to as Assessment and known as Benefit-risk evaluation, it is the review of scientific data in support of the proposed indication of the Product, conducted by a Reviewer/Assessor
Benefit-risk balance	Also known to as Benefit-risk profile or outcome, it is the expert opinion cumulative of the consideration of the benefits and risks - weighing the relative contribution and the uncertainties of the evidence provided, incorporating the current medical knowledge and experience - and recommending a positive or negative outcome

Company/Sponsor	Refers to the owner of the Product, and whom initiates the Submission
Comparator	An investigational or marketed product (i.e. active control) used as a reference in a clinical trial.
Effect size	The quantum of difference arising from the comparison between treatment outcomes of the Product with the comparator; it contributes to the overall interpretation of effectiveness and clinical relevance
Investigated product	Also referred to as the Product, it is the entity on which the Submission of an application for market authorization is based, and for which the clinical studies are conducted
Medicines	For the purpose of this Template, this refers to pharmacological products for use in human with the intention of medical intervention
Patient reported outcomes	Observations as part of a study related to the results obtained directly from the patients, which may include patients' satisfaction, tolerability, symptoms, patient preferences, quality of life and interruptions to daily living
Proforma	Part of the Template; consist of various sections providing the details of the basis on benefit-risk balance decisions
Reviewer	Also known as evaluator or assessor, personnel trained in the scientific evaluation of data, and using clinical judgment to provide a recommendation on the benefit-risk balance of the Product
Risk	Also known as harm, an unfavourable effect or adverse reactions/effects on patients' health, public health or the environment resulting from exposure to the Product**
Seriousness (of adverse event/reaction/effect)*	A serious adverse event (experience) or reaction is any untoward medical occurrence that at any dose:<br><br>• results in death,<br>• is life-threatening (at risk of death at the time of the event)<br>• requires inpatient hospitalisation or prolongation of existing hospitalisation,<br>• results in persistent or significant disability/incapacity, or<br>• is a congenital anomaly/birth defect.

Severity (of adverse event/reaction/effect)*	The intensity of a specific adverse event which may or may not be of medical significance or seriousness, which is defined by a set of criteria.
Submission	An application sent for review to the regulatory authorities by the Company, for the market authorization of the proposed indications of the Product
Summary: Benefit-Risk	Part of the Template; consist of the conclusions of various aspects of assessment, and the final benefit-risk balance
Template	Refers to the entire document comprising the Summary and Proforma
Valuing	An exercise of providing qualitative or quantitative figure (values) reflecting of the effect observed from the studies; this assist in the interpretation of effect size and relevance of treatment
Weighting	An exercise of expert judgment indicating the relative importance of the available options, commonly done through a logical system of rank assignment (weights)

*Adapted from ICH Harmonised Tripartite Guideline. E2A – Clinical Safety Data Management: Definitions and Standards for Expedited Reporting. October 1994.
**Adapted from European Medicines Agency (EMA). Benefit-risk methodology project. Work Package 2 report: Applicability of current tools and processes for regulatory benefit-risk assessment; August 2010.

Completing the Template – Cover page

The Cover page is meant to provide basic information of the Product for which this assessment will be based on.

Compound Identifier(s):	Refers to the entity used during the product development and clinical studies. There may be more than one compound identifier as a result of multiple studies, but they should refer to the same compound being studied for the proposed indication.
Product name/ Brand name / Generic name:	Product name or Brand name - Refers to the entity proposed by the company for market authorization and is considered a trademark, proprietary or commercial entity.<br><br>Generic name – Refers to the entity other than the Product or Brand name which identifies the product and may be an official non-proprietary name of the medicine.
Active Ingredient(s)/ Strength(s)/ Dosage form:	Refers to the pharmacological component of the product; information on strength and dosage form should be included.<br><br>For products with more than one active ingredient, each component should be listed as appropriate.<br><br>For products with multiple strengths, all strengths should be listed.
Proposed Indication:	Refers to the original proposed indication included in the submission. This does NOT represent the recommended indication as a result of the completed assessment. Any amendments to the proposed indication should be presented in Section 7 Conclusions.<br><br>For submissions with multiple proposed indications, a separate assessment using a new template for each indication is required.

Completing the Benefit Risk Summary template

The Summary provides the conclusions of various aspects of benefit-risk assessment, as well as the resulting benefit-risk balance. It is used as a succinct document to communicate the essential decisions for the submission.

Clarifications are provided for selected subsections to guide the user in putting in the correct information.

Note: Subsections which do not currently have any clarifications attached is due to none being raised. Following your use of this template, comments and further clarifications thought to be required are welcomed so that these can be included in the next iteration of the user manual.

Summary 1 .1 Background (Decision Context)

Summary 1.1 Background (Decision Context):

Summary 1.1.1 Specify the proposed therapeutic indication

The proposed indication here refers to the one listed on the cover page.

Summary 1.1.2 Treatment modalities evaluated in this submission

This refers to the overall management of the medical condition as expressed in the proposed indication, including supportive care and current available treatment options considered.

Summary 1.1.3 Is this product for an unmet medical need? Please select

Reason: Please provide justification for your decision on the product fulfilling or not fulfilling an unmet medical need

Summary 2.1 Overall Summaries

The Quality Conclusion accounts for the issues observed during assessment of the quality of the product that may impact the efficacy and safety. Comments should be provided in the instance where there are significant concerns amounting to potential negative consequences in clinical outcomes.

Please tick this box if there are NO findings from the quality assessment that may impact the safe and effective use of the product.

**Summary 2.1.1** Quality Conclusion:	
If box ticked - No relevant findings for the clinical benefit-risk assessment ☐	
If there are relevant findings please comment	If there are SIGNIFICANT findings, please enter these into the box and the potential implications on the safe and effective use of the product

The Non-Clinical Conclusion accounts for the issues observed during the assessment of non-clinical data that may impact the efficacy and safety in humans. Comments should be provided in the instance where there are significant findings & their potential implications for the safe & effective use of the product in humans.

If there are SIGNIFICANT findings, please enter these into the box as well as the potential implications for the safe and effective use of the product in humans.

Please tick this box if there are NO findings from the non-clinical assessment that may impact the safe and effective use of the product in humans.

Summary 2.1.2 Non-Clinical Conclusion:

If box ticked - No relevant findings for the clinical benefit-risk assessment ☐

If there are relevant findings please comment

Summary 2.1.3 Human Phamacology Conclusion:

Only the important results and issues that have an impact on the benefit-risk balance should be described. In addition, unresolved issues or uncertainties should be identified and their impact on the balance assessment should be clearly stated. This includes Bioequivalence, Pharmacokinetic and Dynamic profile, as well as PK, & PD interactions, special populations, dose findings etc.

Summary 2.1.4 Clinical Conclusion:

Only the important results and issues that have an impact on the benefit-risk balance should be described. In addition, unresolved issues or uncertainties should be identified and their impact on the balance assessment should be clearly stated. This includes study design, dosage, population and comparators.

Summary 3.1 Identified Benefits and Risks

List all **benefits** of treatment for this indication as inferred in the submission	Please tick here if Benefit Identified by Reviewer but not by company	Please indicate which **benefits** you believe are justified to be included in the benefit risk assessment by ticking the box	Please explain your main reason **for inclusion or exclusion** of the benefit parameter
	☐	☐	

From the list of all benefits and risks identified in the submitted studies, the reviewer should differentiate those he has identified but not observed by the company. This allows any additional benefits or risks to be highlighted from the reviewer's perspective.

Benefits and risks of treatment should include those observed & derived from the submitted studies as indicated by the Sponsor as well as those identified by the reviewer.

Reasons must be provided for all listed benefits and risks as to their inclusion or exclusion for further benefit-risk assessment.

Uncertainties of the identified benefits and risks will be addressed in summary 6.1.

After a review of the list of identified benefits and risks, the reviewer should decide which are pivotal in making the benefit-risk balance. For each benefit or risk justified to be included, these would be auto-populated respectively to summary 6.1, where detailed information will then be further required.

Summary 3.1.2 Risks documented

List all **risks** of treatment for this indication as inferred in the submission	Please tick here if Risk Identified by Reviewer but not by company	Please indicate which **risks** you believe are justified to be included in the benefit risk assessment by ticking the box	Please explain your main reason **for inclusion or exclusion** of the risk parameter
	☐	☐	

Summary 4.1 Clinical Study Summary

Study Ref. Type	Study Design (N)(duration) R, C, DB, OL (N=)(weeks/months) ·Non-inferiority/Superiority/ Observational study ·State primary objective ·State primary efficacy parameter	Treatment ·Treatment arm Active (name, dose, freq, duration) ·Comparator arm Placebo / Active (name, dose, freq, duration)	Conclusion ·Results of primary efficacy parameter ·Results of other relevant efficacy endpoints ·Conclusion of study (outcomes, strength of study, weight of evidence, and clinical significance)	
				-

List the statistical parameter applied to investigate the endpoint used to confirm the benefit, or in the case of non-inferiority and equivalence studies, the pre-defined margins or deltas not to be exceeded.

Summary 5.1 Risks: Overall Summary

Table of pooled overall incidence of events can be added below

Adobe **Acrobat** users can click here to attach a file: Attach a file *(Note: this will not activate in Adobe **Reader**)*

Click in the space below to upload an image: (jpeg, gif, png): *(Available to both Adobe Reader and Acrobat users)*

Summary 6.1 Weights and values

Benefits	Relative Importance (weighting)	Valuing the options			Comment on strength and uncertainty of benefit
		Investigated product	Comparator	Placebo	

Risks	Relative Importance (weighting)	Valuing the options			Comment on strength and uncertainty of each risk	Was the value or weight of this risk altered or mitigated by the ability to control the use of the medicine once on the market?
		Investigated product	Comparator	Placebo		

- *Assigning Relative Importance*

For the identified benefits and risks, the reviewer should apply their expert judgment to provide relative importance of each parameter in contributing to the benefit-risk balance, in the light of the evidence provided

This is either carried out through ranking, numerical value, or qualitative descriptors such as high, medium or low. If the system of ranking is used, then this should be hierarchical and logical. Reviewers should limit themselves to using only one of these systems for both benefits and risks.

- *Valuing*

Provide either qualitative (e.g. high, medium, low or absent) or quantitative (utilizing the values from the study outcomes, e.g. overall survival 32% for product versus 27% for placebo) values of benefits and risks. When possible, please use quantification.

A short explanation of the selected systems for assigning relative importance and valuing should be provided in the allocated box:

Please describe methodology used for assessing relative importance: eg Ranking or point allocation and also what is has been used in relation to valuing the options e.g. % change, Number of patients, etc

Summary 7.1 Conclusion

The final decision on the benefit-risk balance of the Product for the proposed indication will be discussed here. Considerations for assessing the benefit-risk balance are adapted from the EMA reflection paper (2008) and these are attached in Appendix A, B, and C.

**Summary 7.1.1** If the benefit-risk balance is assessed to be negative, describe the harm (e.g. in terms of lack of efficacy, toxicity) that the drug may cause if used in the proposed indication
**Summary 7.1.2** Describe how the benefit-risk balance is expected to evolve over time (e.g. when late side effects emerge or long-term efficacy decreases)
**Summary 7.1.3** Describe outstanding issues, and other significant information eg, submission of additional reports by the company to address those issues, hearings and advisory group recommendations, information from other jurisdictions (eg advisory committees, scientific experts, patients, consumers, consumer advocates and other stakeholders)
**Summary 7.1.4** Make reference to the evaluation of the pharmacovigilance plan and risk minimization plan if any. Describe any communication or particularly significant information to the medical profession, patients or the public that is required. Describe restrictions to product availability or usage
**Summary 7.1.5** Describe the need for further studies (e.g. the need for studies to improve the benefit-risk balance with further optimization studies, the need for intensive additional follow up measures or specific obligations, and the need for further development including any paediatric development plans.
This section should also consider any further studies required to mitigate findings from non-clinical studies (Section 2.2.2).

Summary 7.1.6 Please provide any other information considered by the agency relevant to the benefit risk decision that is not covered elsewhere in the proforma.

Summary 7.1.7 Please provide a clear conclusion on the benefit-risk being positive or not for the proposed indication.

The conclusion here refers to the benefit-risk balance of the Product for this proposed indication, and does not constitute a final regulatory decision.

Summary 7.1.8 Please provide the indication recommended following the outcome of the benefit-risk balance.

Amendments should include all changes to proposed indication and/or dosing regimen, with annotations provided. Please provide justification for the amendments.

For negative benefit-risk balance resulting in a recommendation for non-approval, kindly indicate so.

These recommendations do not necessarily constitute final regulatory decision or market authorizations.

Reviewers Name:

Signature:

Date:

Manager sign-off or Peer review

Reviewers Name:

Signature:

Date:

Navigating through the template

Hyperlinks are provided at the Table of Contents to help locate the desired section.

Table of Contents		
**Benefit-Risk Summary**		
1.1	Background (Decision Context)	Go to Page
2.1	Overall Summaries	Go to Page
3.1	Identified Benefits and Risks	Go to Page
4.1	Clinical Study Summary	Go to Page
5.1	Table of Pooled overall Incidence of events	Go to Page
6.1	Relative Importance and Values	Go to Page
7.1	Conclusion	Go to Page

Thumbnails are available at the left side of the screen panel to help navigate to the desired page.

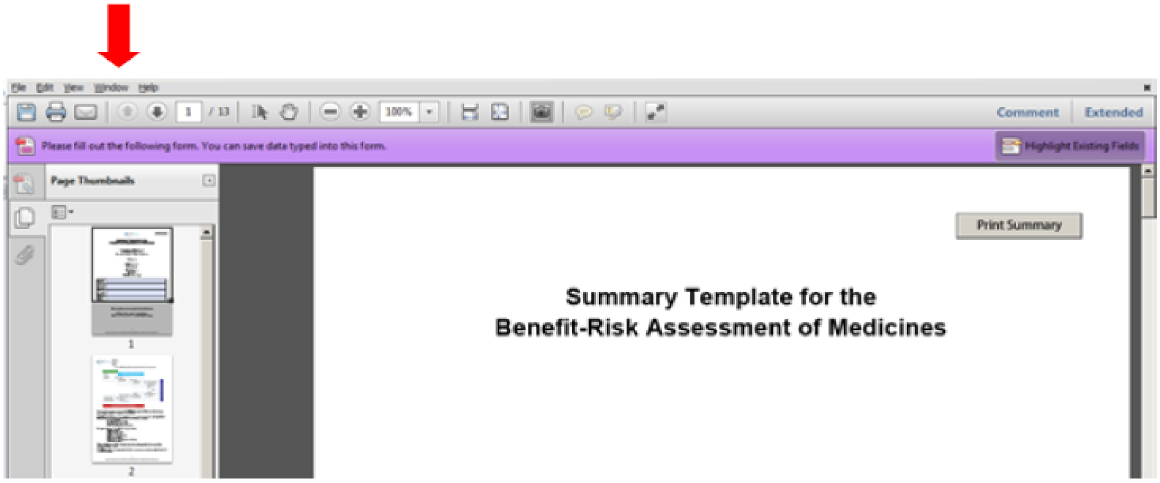

Appendix A

Criteria for assessing efficacy or favourable effects*

1. Efficacy (primary endpoint) versus comparator and its clinical relevance
2. Magnitude of treatment effect
3. Clinical relevance of the primary endpoints
4. Statistical significance of the efficacy results
5. Representiveness of the studied population for the population targeted in the label
6. Discussion of dose
7. Evidence for the efficacy in relative subgroups
8. Design conduct and statistical adequacy of the trial
9. Confirmation of treatment effect by results of non-primary endpoints
10. Validation of scales and outcome measures
11. Patient preferred outcomes
12. Confirmation of efficacy by results of relevant non-pivotal trials and extensions
13. Anticipated patient compliance (and patient convenience)
14. Clustering (consistency) of results of the pivotal trials

*Adapted from European Medicines Agency (EMA). Reflection Paper on Benefit-risk Assessment Methods in the context of the Evaluation of Marketing Authorisation Applications of Medicinal Products for Human Use; March 2008.

Appendix B

Criteria for assessing harms or unfavourable effects*

1. Overall incidence of adverse effects (from clinical trials)
2. Overall incidence of serious adverse effects (from clinical trials
3. Discontinuation rate due to adverse effects (from clinical trials)
4. Incidence, seriousness and duration of specific adverse effects (from clinical trials and post-marketing surveillance)
5. Interaction with other drugs and food
6. Safety in subgroups (e.g. race and sex)
7. Potential for off label use leading to safety hazards
8. Potential for non-demonstrated additional risk due to limitations of clinical trials and/or short market exposure.
9. Potential for non-demonstrated additional risk due to safety issues observed in pre-clinical safety studies but not in humans
10. Potential for non-demonstrated additional risk due to safety issues observed with other medicines of the same pharmacological class

*Adapted from European Medicines Agency (EMA). Reflection Paper on Benefit-risk Assessment Methods in the context of the Evaluation of Marketing Authorisation Applications of Medicinal Products for Human Use; March 2008.

Appendix C

Criteria for assessing benefit-risk balance*

- Amount of evidence to characterise the benefit-risk balance:
  - Availability of comparative data and their limitations and potential deficiencies
- Interpret of key benefits and risks
  - from perspectives of different stakeholders, including patients and treating physicians
- Level of risk acceptability
  - corresponding to the perceived degree of clinical benefit in the specific context
- Relating the benefits to the risks when possible:
  - Using logical comparisons e.g. potential lives saved as a result of treatment compare to potential lives lost as a result of adverse reactions
- Factors affecting the benefit-risk balance:
  - Situations that may alter the current balance e.g. different patient or disease characteristics
- Sensitivity of the benefit-risk balance:
  - Discussion on the potential changes to the balance if the fundamental assumptions are to be amended
- Other appropriate discussions:
  - Effectiveness of proposed treatment compared to available options
  - For negative benefit-risk balanced, describe the potential harm incurred upon exposure for the claimed indication
  - Evolution of benefit-risk balance over time
  - Outstanding issues, submission or reports to address identified issues
  - Evaluation of pharmacovigilance plan, risk mitigation plan or other post-marketing commitments including need for further studies
  - Opinions from scientific experts, patients, consumers or advocates and other stakeholders in the benefit-risk assessments
- Conclusion on the benefit-risk being positive or not for every claimed indication.

*Adapted from European Medicines Agency (EMA). Reflection Paper on Benefit-risk Assessment Methods in the context of the Evaluation of Marketing Authorisation Applications of Medicinal Products for Human Use; March 2008.
