# Supplementary material for: A practical approach to communicating benefit-risk decisions of medicines to stakeholders
Source: Front Pharmacol. 2015 Jun 11;6:99. doi: 10.3389/fphar.2015.00099 (PMC4463867; doi:10.3389/fphar.2015.00099)
Supplement: Supplementary file 3 [file Presentation3.PDF]

Appendix C

Evaluation of the use of the Benefit-risk Summary

Professor Sam Salek, Dr James Leong and Professor Stuart Walker

Confidentiality

- All information collected will be kept strictly confidential.
- No data that will identify a participant will be reported, or details made available to a third party.
- External reports or presentations of the data will include only anonymous figures and any appropriate analytical interpretation.
- Data will only be provided to the relevant organization concerned.

Instructions for completion of the tool

This tool relates to your recent experience with the following system:

Benefit-risk_Summary_2013

There are 3 sections:

A. User-friendliness
B. Documentation
C. Applicability

This tool should be completed as soon as the documentation of the benefit-risk assessment using the Summary is completed.

You should relate each statement to your experience of using the Summary and tick the box that best describes your opinion.

We would appreciate if you could provide a response to all the items and submit the completed form by ……………… Please provide your responses electronically and note that the comment boxes are expandable.

Your comments are extremely valuable, please feel free to use the boxes provided for this purpose.

A. User-friendliness

A practical summary should be easy to use and understand.

Having used the Summary to document the assessment of benefits and risks, please read through the list of items below and put a tick in the box that best describes your opinion.

Kindly provide comments if your opinion is "Fair" or "Poor".

<table>
<tr><th></th><th></th><th>Excellent</th><th>Good</th><th>Fair</th><th>Poor</th><th>Did not use</th><th>Provide comments if opinion is "Fair" or "Poor"</th></tr>
<tr><td colspan="8">Navigating through the Summary</td></tr>
<tr><td>1</td><td>
<table>
<tr><th colspan="3">Table of Contents</th></tr>
<tr><td colspan="2">Benefit-Risk Summary</td><td></td></tr>
<tr><td></td><td></td><td></td></tr>
<tr><td>1.1</td><td>Background (Decision Context)</td><td>Go to Page</td></tr>
<tr><td>2.1</td><td>Overall Summaries</td><td>Go to Page</td></tr>
<tr><td>3.1</td><td>Identified Benefits and Risks</td><td>Go to Page</td></tr>
<tr><td>4.1</td><td>Clinical Study Summary</td><td>Go to Page</td></tr>
<tr><td>5.1</td><td>Table of Pooled overall Incidence of events</td><td>Go to Page</td></tr>
<tr><td>6.1</td><td>Relative Importance and Values</td><td>Go to Page</td></tr>
<tr><td>7.1</td><td>Conclusion</td><td>Go to Page</td></tr>
</table>
Navigation to required sections using the "Go to Page" button in Table of Contents.</td><td></td><td></td><td></td><td></td><td></td><td></td></tr>
<tr><td>2</td><td>Navigation to required sections using the page thumbnails found on the left side of screen display.</td><td></td><td></td><td></td><td></td><td></td><td></td></tr>
<tr><td colspan="8">Guidance by user manual in completing the Summary</td></tr>
<tr><td>3</td><td>Clarity of instructions.</td><td></td><td></td><td></td><td></td><td></td><td></td></tr>
<tr><td>4</td><td>Comprehensiveness of guidance provided.</td><td></td><td></td><td></td><td></td><td></td><td></td></tr>
<tr><td>5</td><td>Applicability of guidance.</td><td></td><td></td><td></td><td></td><td></td><td></td></tr>
</table>

Please comment on how to further improve the user-friendliness, or suggest other functions that might improve the navigation of the Summary.

B. Documentation

A functional summary should be able to document the processes leading to the final benefit-risk conclusion in a structured and systematic manner.

Having used the Summary to document the benefits, risks and the resulting balance, please read through the list of items below, and put a tick in the box that best describes your opinion.

- "**Fit for purpose**" refers to the Summary being able to achieve the item for the majority of the applications.
- "**Fit for purpose with modification**" refers to the Summary being able to achieve the item with amendments (kindly specify the changes required).
- "**Not fit for purpose**" refers to the Summary not being able to achieve the item at all.

Kindly provide comments as required for your opinion, as indicated in the section.

**Summary 1.1 Background (Decision Context) (includes summaries 1.1.1 to 1.1.3)**		**Fit for purpose**	**Fit for purpose with modification**	**Not fit for purpose**
1	Documents relevant information to support the decision context.		*Specify modification(s) needed:*	*Comment(s):*

**Summary 2.1 Overall Summaries**		**Fit for purpose**	**Fit for purpose with modification**	**Not fit for purpose**
**Summary 2.1.1 Quality Conclusion**				
2	Documents relevant information to support the decision context.		*Specify modification(s) needed:*	*Comment(s):*
**Summary 2.1.2 Non-Clinical Conclusion**				
3	Documents relevant information to support the decision context.		*Specify modification(s) needed:*	*Comment(s):*

Summary 2 Overall Summaries (continued)		Fit for purpose	Fit for purpose with modification	Not fit for purpose
**Section 2.1.3 Human Pharmacology Conclusion**				
4	Documents relevant information to support the decision context.		*Specify modification(s) needed:*	*Comment(s):*
**Section 2.1.4 Clinical Conclusion**				
5	Documents relevant information to support the decision context.		*Specify modification(s) needed:*	*Comment(s):*

<table>
<tr><th colspan="2">Summary 3.1 Identified Benefits and Risks (includes summaries 3.1.1 and 3.1.2)</th><th>Fit for purpose</th><th>Fit for purpose with modification</th><th>Not fit for purpose</th></tr>
<tr><td colspan="5">Benefits
<table><tr><td>List all benefits of treatment for this indication as inferred in the submission</td><td>Please tick here if Benefit Identified by Reviewer but not by company</td><td>Please indicate which benefits you believe are justified to be included in the benefit risk assessment by ticking the box</td><td>Please explain your main reason for inclusion or exclusion of the benefit parameter</td></tr><tr><td></td><td>☐</td><td>☐</td><td></td></tr></table></td></tr>
<tr><td>6</td><td>Documents the reasons for inclusion or exclusion of all the benefits.</td><td></td><td>Specify modification(s) needed:</td><td>Comment(s):</td></tr>
<tr><td>7</td><td>Documents the relevant benefits as identified by the sponsor.</td><td></td><td>Specify modification(s) needed:</td><td>Comment(s):</td></tr>
<tr><td>8</td><td>Documents your list of benefits to be included in the benefit-risk assessment.</td><td></td><td>Specify modification(s) needed:</td><td>Comment(s):</td></tr>
</table>

Risks

List all **risks** of treatment for this indication as inferred in the submission	Please tick here if Risk Identified by Reviewer but not by company	Please indicate which **risks** you believe are justified to be included in the benefit risk assessment by ticking the box	Please explain your main reason **for inclusion or exclusion** of the risk parameter
	☐	☐	

9	Documents the reasons for inclusion or exclusion of all the risks.		*Specify modification(s) needed:*	*Comment(s):*
10	Documents the relevant risks as identified by the sponsor.		*Specify modification(s) needed:*	*Comment(s):*
11	Documents your list of risks to be included in the benefit-risk assessment.		*Specify modification(s) needed:*	*Comment(s):*

Summary 4.1 Clinical Study Information		Fit for purpose	Fit for purpose with modification	Not fit for purpose

Study Ref. Type	Study Design (N)(duration) R, C, DB, OL (N=)(weeks/months) ·Non-inferiority/Superiority/ Observational study ·State primary objective ·State primary efficacy parameter	Treatment ·Treatment arm Active (name, dose, freq, duration) ·Comparator arm Placebo / Active (name, dose, freq, duration)	Conclusion ·Results of primary efficacy parameter ·Results of other relevant efficacy endpoints ·Conclusion of study (outcomes, strength of study, weight of evidence, and clinical significance)	
				-

12	Documents the outcomes and conclusions of the studies.		*Specify modification(s) needed:*	*Comment(s):*

Summary 5.1 RISKS: Overall Summary		Fit for purpose	Fit for purpose with modification	Not fit for purpose

Table of pooled overall incidence of events can be added below

Adobe **Acrobat** users can click here to attach a file: Attach a file *(Note: this will not activate in Adobe **Reader**)*

Click in the space below to upload an image: (jpeg, gif, png): *(Available to both Adobe Reader and Acrobat users)*

13	Documents the overall summary of the incidence of adverse events/effects.		*Specify modification(s) needed:*	*Comment(s):*

Summary 6.1 Weights and Values		Fit for purpose	Fit for purpose with modification	Not fit for purpose
**Benefits**				

Benefits	Relative Importance (weighting)	Valuing the options			Comment on strength and uncertainty of benefit
		Investigated product	Comparator	Placebo	

For items 14 and 15, please refer to the table above.

		Fit for purpose	Fit for purpose with modification	Not fit for purpose
14	Documents the contribution of the weighting/relative importance of the benefits to the final benefit-risk decision.		*Specify modification(s) needed:*	*Comment(s):*
15	Documents the contribution of the values of the benefits from the studies to the final benefit-risk decision.		*Specify modification(s) needed:*	*Comment(s):*

Risks

Risks	Relative Importance (weighting)	Valuing the options			Comment on strength and uncertainty of each risk	Was the value or weight of this risk altered or mitigated by the ability to control the use of the medicine once on the market?
		Investigated product	Comparator	Placebo		

For items 16 and 17, please refer to the table above.

		Fit for purpose	Fit for purpose with modification	Not fit for purpose
16	Documents the contribution of the weighting/relative importance of the risks to the final benefit-risk decision.		*Specify modification(s) needed:*	*Comment(s):*
17	Documents the contribution of the values of the risks from the studies to the final benefit-risk decision.		*Specify modification(s) needed:*	*Comment(s):*

Summary 7.1 Conclusion (includes summaries 7.1.1 to 7.1.8)		Fit for purpose	Fit for purpose with modification	Not fit for purpose
18	Includes all the relevant information to draw a conclusion regarding the recommendation.		*Specify modification(s) needed:*	*Comment(s):*

C. Applicability

The summary used to document the benefit-risk balance should contribute significantly and apply directly to the communication of regulatory decision making.

Having used a structured systematic documentation of benefits and risks assessment, kindly indicate if this Summary should be part of standard regulatory review practices.

		Excellent	Good	Fair	Poor	Provide comments if opinion is " Fair" or "Poor"
1	The Summary's contribution to promoting effective communication to stakeholders.					
2	The Summary's contribution to achieving consistency of decisions between regulatory agencies.					
3	The Summary's advantages over the systems I am currently using in my organisation.					

		Yes	No (please provide comments)
Irrespective of the jurisdiction in your country, are you willing to share the entire Summary with the following stakeholders?<br><br>If no, please provide a reason.			
4	• Healthcare professionals		
5	• Health technologies assessment agencies (HTA)		
6	• Patients/patient advocacy groups		
7	• Other regulatory agencies		
8	• Media/public domain		
9	• Academia		

If you have any further comments, kindly use the space below:

Should you have further questions or concerns about this tool, please contact:

……………………………………………………………

On completion, please send this to the following email:

……………………………………………………………

Participant's Information

**Name/Signature:**	**Position:**
**Date:**	

---- End ---
